# Does ozaki procedure have a future as a new surgical approach for aortic valve replacement? a systematic review and meta-analysis

**DOI:** 10.1097/MS9.0000000000000982

**Published:** 2023-07-17

**Authors:** Ahmed K. Awad, Ramadan A. Farahat, Eman Reda Gad, Mahmoud Shaban Abdelgalil, Aly Sherif Hassaballa

**Affiliations:** a Faculty of Medicine; b Assistant Lecturer of Cardiothoracic Surgery Department, Faculty of Medicine, Ain Shams University; c Faculty of Medicine, Cairo University, Cairo; d Faculty of Medicine, Kafrelsheikh University, Kafrelsheikh, Egypt

**Keywords:** aortic valve, meta-analysis, Ozaki procedure, review

## Abstract

**Background:**

In 2014, Ozaki *et al.* introduced the neo-cuspidation (Ozaki procedure), a new valve from the pericardium, to reduce or even prevent the risk of chronic autoimmune inflammation and subsequent rejection or valve degeneration. Thus, the authors aimed to assess the safety and efficacy of the Ozaki technique in treating aortic valve diseases.

**Materials and methods:**

A comprehensive search was performed via PubMed, the Cochrane Library, Scopus, and the Web of Science up to 20 February 2022. Random-effects meta-analysis models were employed to estimate the pooled mean and SD or event to the total of the Ozaki procedure. Relevant records were retrieved and analyzed by OpenMeta analyst software.

**Results:**

A total of 2863 patients from 21 studies were finally included in our analysis. Ac. Ozaki technique showed statistical significance in terms of mean cardiopulmonary bypass time of 148 mins (95% CI 144–152.2, *P*<0.001), mean aortic cross-clamp time of 112.46 mins (95% CI 105.116, 119.823, *P*<0.001), reoperation with a low risk of 0.011 (95% CI 0.005, 0.016, *P*=0.047), conversion to aortic valve replacement with a low risk of 0.004 (95% CI −0.001, 0.008, *P*=0.392), finally ICU stay (days) and hospital length of stay (days) with a mean of 2.061 days (95% CI 1.535, 2.587, *P*<0.001) and 8.159 days (95% CI 7.183–9.855, *P*<0.001), respectively.

**Conclusion:**

The Ozaki procedure provides a safe surgical technique with low mean cardiopulmonary bypass time and aortic cross-clamp time; moreover, a mean of 2-day-postoperative hospital stay was observed with the Ozaki procedure with a low risk of conversion to aortic valve replacement, reoperation, ICU and hospital stay, and death.

## Introduction

Highlights

- The Ozaki procedure was first introduced by Ozaki *et al.* in 2014.
- The Ozaki procedure is a new surgical approach for aortic valve replacement.
- Our meta-analysis highlights the safety of the Ozaki procedure in terms of its low cardiopulmonary bypass time and aortic cross-clamp times.
- The Ozaki procedure has a mean of 2 days hospital stay postoperatively, which is the lowest compared to other known valve replacement modalities.
- Ozaki has a low risk of both aortic valve reoperation and conversion to aortic valve replacement.

Being the main affected valve in the human heart, aortic valve diseases constitute a large public health burden. Aortic valve diseases are either aortic stenosis (AS) or regurgitation (AR), which both have detrimental effects on the cardiac muscle and systemic circulation. AS is evident in the elderly population and its incidence increases with age to a prevalence of 9.8% of the population in the eighth causing a variety of clinical manifestations such as left ventricular hypertrophy, decrease cardiac output, and heart failure^1,2^. On the other hand, AR decreases with age with a prevalence of 4.9%, which is increasing with age until the sixth decade when incidence decreases, yet the percentages may be underestimated due to the AS-associated degree of AR^3^.

Treatment of valvular heart disease accounts for 10–20% of all cardiac surgical procedures performed in the USA, with aortic valve replacement (AVR) accounting for around two-thirds of all heart valve surgeries^4^. The gold standard in treating aortic valve disease is AVR using another valve with the firstly introduced mechanical valves (MV), then biological or prosthetic valves^5^. Both have their advantages and disadvantages with favor biological valves due to no need for hazardous anticoagulants and more durability. Nevertheless, biological valves are synthesized from bovine or pig pericardium, which causes an unavoidable state of chronic autoimmune inflammatory response that may lead to degeneration and valve replacement^5^. Therefore, in 2014, Ozaki *et al.* ^6^ introduced a new surgical technique which is neo-cuspidation; forming a new valve from the pericardium decreasing the risk of inflammation and rejection. Thus, we aimed in our systematic review and meta-analysis to further assess the safety and efficacy of the Ozaki technique in treating aortic valve diseases.

## Materials and methods

The Preferred Reporting Items for Systematic Reviews and Meta-Analyses (PRISMA) guideline and Cochrane handbook were fulfilled in this systematic review and meta-analysis^7,8^. Our meta-analysis was registered on the research registry with an identification number: researchregistry9010.

### Search strategy

We searched PubMed, the Cochrane Library, Scopus, and the Web of Science for studies that investigate the usage of the Ozaki procedure for patients who need AVR up to 20 October 2022. The following search terms were used: ((Ozaki technique OR Ozaki procedure OR aortic valve neo-cuspidization) AND (Aortic valve regurgitation OR aortic valve insufficiency OR Aortic Valve Incompetence)); moreover, reviewing the reference lists of retrieved articles were used to complement the broad search.

### Eligibility criteria

Studies that investigate the usage of the Ozaki procedure for AVR and articles that were published in peer-reviewed international journals and had enough data for qualitative and quantitative analysis were included with no language restriction. We excluded conference papers, unpublished articles, letters to the editor, posters, and animal studies.

### Data extraction

We extracted the following data from the included studies as baseline characteristics: name of the First author, publication year, country, study design, sex, mean age, total sample size, BMI, BSA, cardiovascular risk factors, aortic valve pathology, and aortic valve anatomy (uni, bi, tri, quad cuspid) (Table 1). Furthermore, for qualitative and quantitative analysis, we extracted mean cardiopulmonary bypass time (min) (CPBT), mean cross-clamp time (min), reoperation, converted to AVR, ICU stay days, hospital length of stay (days), mean aortic valve mean gradient (mmHg), peak aortic valve pressure gradient mmHg, and death.

**Table 1:** Baseline characteristics of included studies

							Cardiovascular risk factors		Aortic valve pathology		Aortic valve anatomy (uni, bi, tri, quad cuspid)			
References	Sample size	Study design	Age, years, Mean (SD)	sex (Male)	BMI, mean, SD	BSA, mean (SD)	DM	HTN	AS	AR	Uni	Bi	Tri	Quad
Ozaki 2014^6^	404	PC	69 (12.9)	201	NA	NA	NA	NA	289	115	NA	NA	NA	NA
Sergio 2021	71	RC	52 (14.2)	52	NA	NA	5	26	33	NA	NA	13	58	NA
Sheng 2019	36	PC	46.7 (16.6)	19	25.52 (4.9)	1.71 (1.2)	5	15	NA	NA	NA	10	26	NA
Shigeyuki 2015	416	RC	71.2 (12.0)	182	NA	NA	NA	NA	NA	NA	16	114	286	NA
Shigeyuki 2018	850	RC	71 (7.2)	444	NA	NA	NA	NA	534	254	28	224	596	2
Yamamoto 2016	23	RC	72.1 (5.5)	4	NA	1.57 (0.11)	1	8	NA	NA	NA	NA	NA	NA
Polito 2021	38	PC	18	NA	43-73^*^	1.9 (1)	NA	8	20	NA	5	11	5	NA
Ozaki 2014^6^	108	PC	11.2-47.8^†^	75	NA	NA	NA	NA	51	43	11	57	NA	NA
Oliver 2018	30	PC	10.55-66.83^†^	20	NA	NA	3	NA	7	12	NA	6	24	NA
Nguyen 2018	9	PC	34 (12)		NA	NA	NA	17	3	1	NA	3	NA	NA
Ngo 2020	61	RC	55.8 (13.0)	41	NA	NA	1		24	17	NA	16	NA	NA
Krane 2020	103	PC	54.0 (16.4)		29.9 (5.4)	2.0 (0.2)	12	30	80	23	NA	81	NA	NA
Kawase 2012	304	PC	48.9 (19.9)	8	NA	NA	NA	55	8	7	9		NA	NA
Baird 2020	57	RC	1–18^†^	21	44.3^*^	1.33	NA	NA	33	52	9	20	26	2
Ozaki 2014^6^	86	PC	82.9 (2.5)	27	NA	NA	NA	NA	72	14	NA	8	NA	NA
Roussakis 2021	1	Case report	21	1	NA	NA	NA	NA	1	NA	1	0	0	0
Angelo Politoa, 2020	22	PC	13.3 (5.07)	NA	54 (30.91)^*^	NA	NA	NA	10	12	5	11	5	NA
Luke 2019	58	RC	14.07 (4.71)	NA	51.4 (26.53)^*^	NA	NA	NA	NA	NA	8	34	15	1
Marathe 2020	51	RC	7.9 (6.07)	36	21 (16.25)	1 (0.42)	NA	NA	23	22	12	15	23	1
Marathe 2021	33	RC	9.3 (6.075)	26	29.2 (16.8)	1 (0.37)	NA	NA	3	17	15	9	9	0
Shigeyuki Ozaki 2014	102	PC	63.7 (10.0)	55	NA	NA	NA	NA	77	25	NA	NA	NA	NA

* Weight in kg, mean, and (SD).

† Age reported as a range.

AR, aortic regurgitation; AS, aortic stenosis; DM, Diabetes mellitus; HTN, hypertension; NA, not available; PC, prospective cohort; RC; retrospective cohort.

### Quality assessment

We used the Newcastle–Ottawa Scale (NOS) to assess observational studies^9^. The domains of NOS include three domains for selection, comparability, and outcomes: Representativeness of the Exposed Cohort, Selection of the Non-Exposed Cohort, Ascertainment of Exposure, Demonstration that Outcome of Interest Was Not Present at Start of Study, Comparability of Cohorts based on the Design or Analysis, Assessment of Outcome, Was Follow-Up Long Enough for Outcomes to Occur, and Adequacy of Follow-Up of Cohorts. Two independent reviewers (R.A.F and M.S.A) screened the methodological quality of the included studies, and in the case of discrepancies, they were resolved by a senior author. Moreover, the work has been outlined in accordance with AMSTAR (Assessing the Methodological Quality of Systematic Reviews) Guidelines^10^, Supplemental Digital Content 1, http://links.lww.com/MS9/A174.

### Data-analysis

We conducted a single-arm meta-analysis using OpenMeta analyst software^11^. Random-effects meta-analysis models were employed to estimate the pooled mean and SD or event to the total in the form of risk (R) in the Ozaki procedure. A *P*-value less than 0.05 was considered statistically significant. The data was continuous, we used the mean and risk with a 95% CI to assess the estimated effect measure. Moreover, heterogeneity was evaluated using the inconsistency (*I*^2^) and *χ*^2^ tests. *I*^2^>50% was considered substantial heterogeneity in the studies. If substantial heterogeneity was found, a leave-one-out meta-analysis was performed to show how each study affects the overall estimate by excluding one study interchangeably at a time from the meta-analysis.

## Results

### Search results

**Figure 1:**
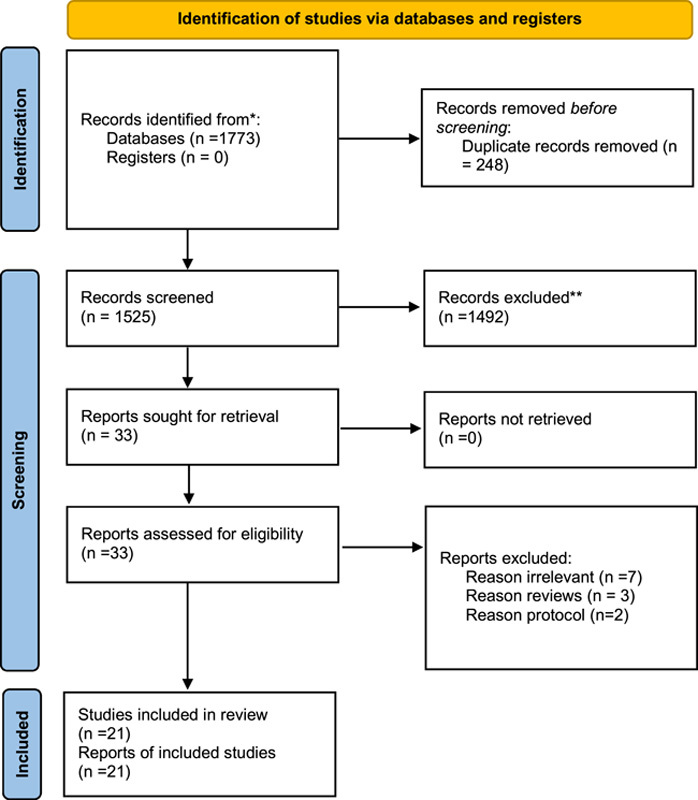
Preferred Reporting Items for Systematic Reviews and Meta-Analyses flow diagram.

Our search strategy resulted in a total number of 1525 studies. After the title and abstract screening and removing the duplicates, 33 full-text articles were evaluated for eligibility. According to the PRISMA checklist, we performed the full-text screening with 21 papers met our criteria and were included in our meta-analysis (Fig. 1, Supplementary Table 1, Supplemental Digital Content 2, http://links.lww.com/MS9/A175). Nine studies were retrospective cohorts, 11 were prospective cohorts, and one was case report.

### Summary of the included studies

With a mean age of 35.5 (15.8), Our study included 2863 participants of which 1212 patients were males. AS was the indication of AVR in 979 patients, while AR was in 499 patients with the rest being either unknown or a mix of both. The mean BMI is 25.24 and the mean BSA is 1.5. The baseline characteristics are illustrated in Table 1.

### Quality assessment

For our included observational studies (9 retrospective cohorts, 11 prospective cohorts) studies, judged by following NOS guidelines, 14 were of good quality and 6 were of fair quality mostly due to the nonmatching of the cases and controls regarding the confounders and lack of selection of controls as shown in (Supplementary Table 2, Supplemental Digital Content 3, http://links.lww.com/MS9/A176). Furthermore, according to AMSTAR-2 guidelines, our study was compliant with the guidelines and scored high confidence of evidence according to the checklist (Supplementary Table 3, Supplemental Digital Content 4, http://links.lww.com/MS9/A177).

### Data-analysis

#### Operative analysis

**Figure 2:**
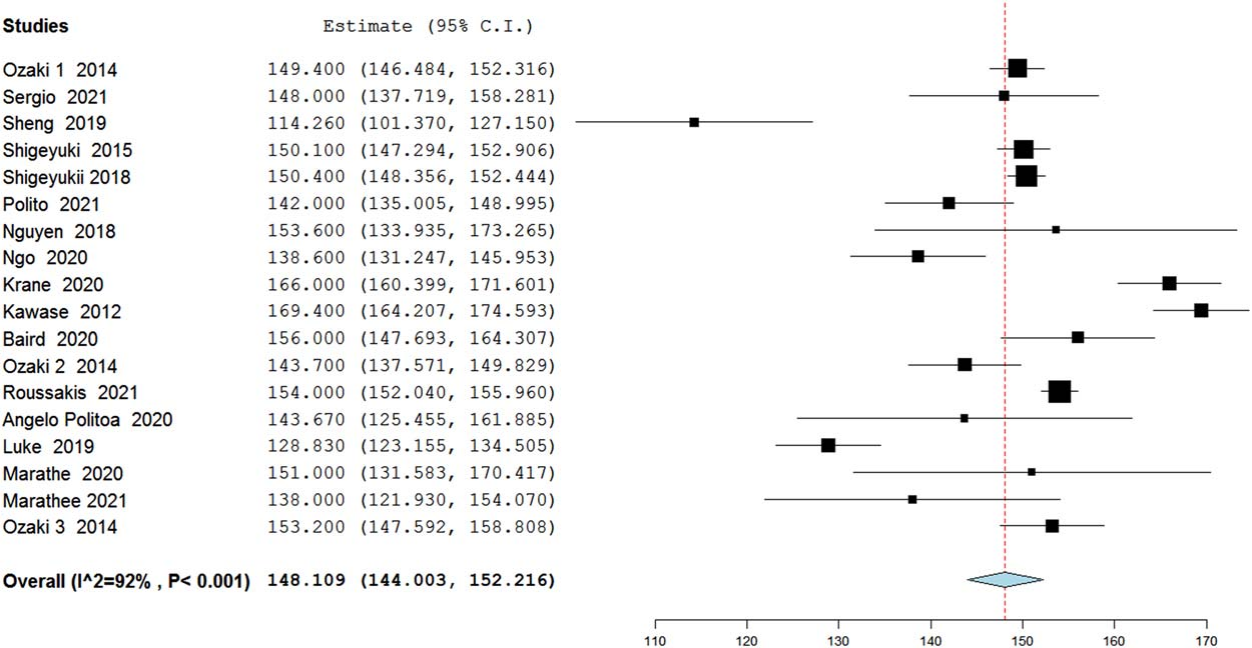
Forest plot of mean cardiopulmonary bypass time (min) analysis.

**Figure 3:**
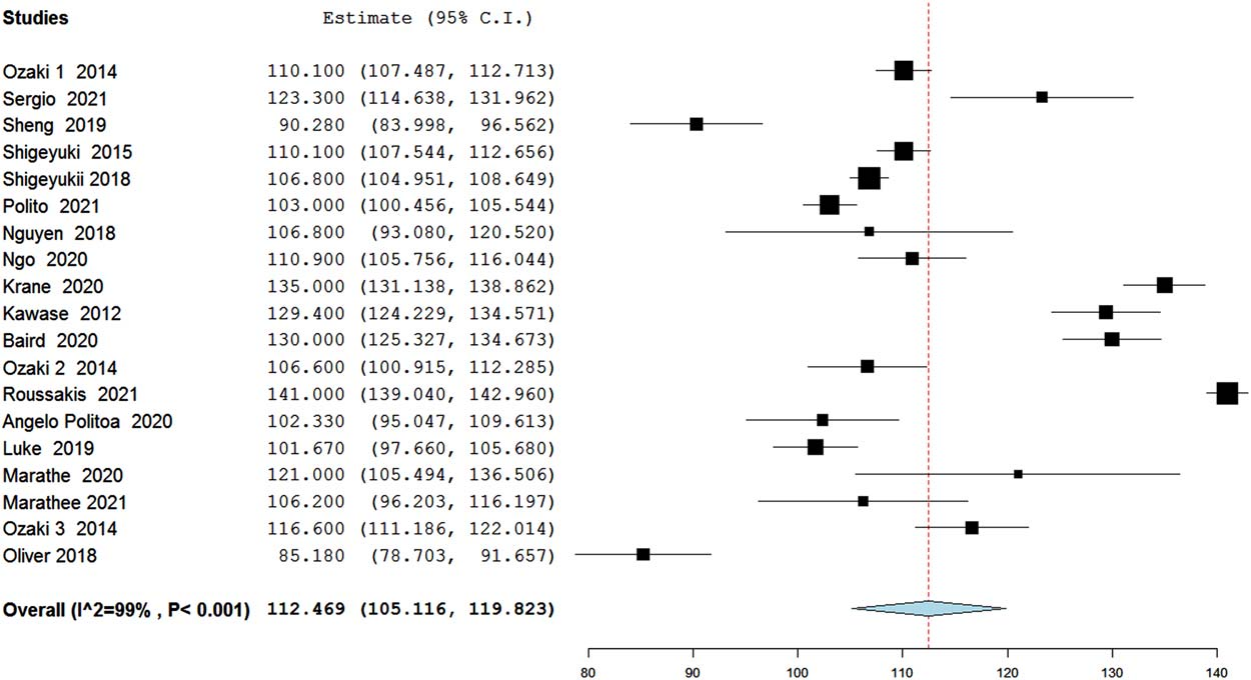
Forest plot of mean aortic cross-clamp time (min).

**Figure 4:**
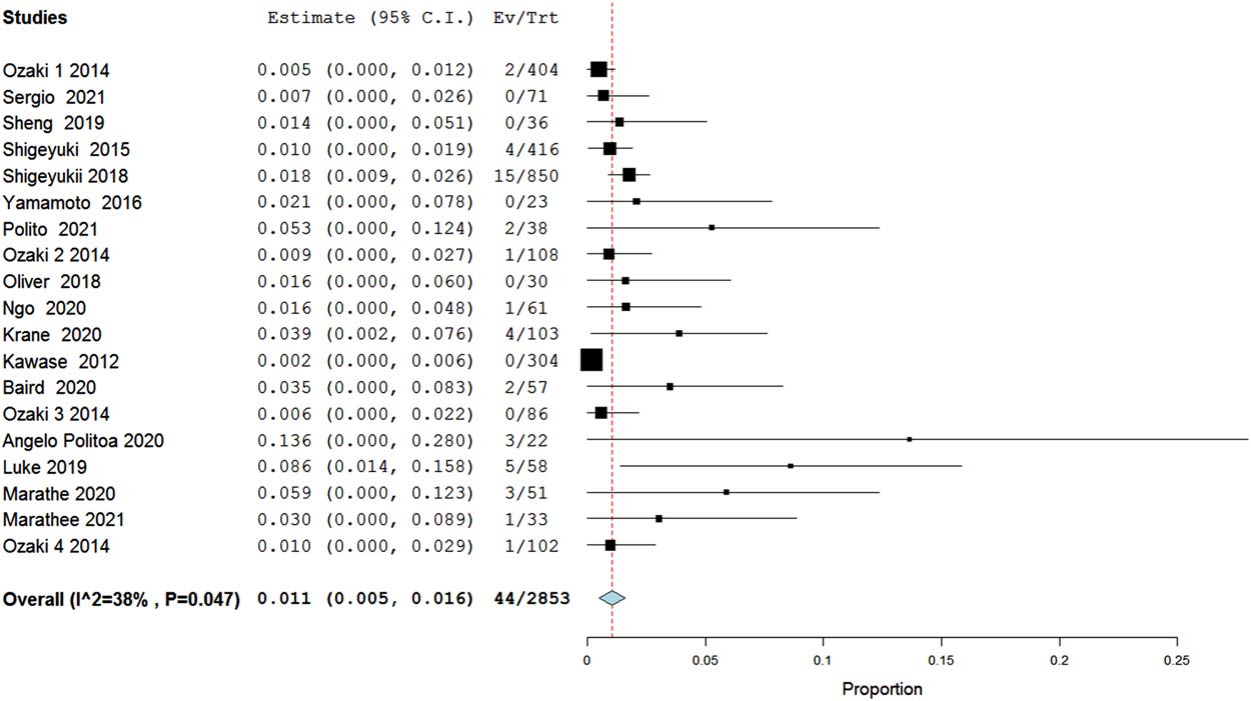
Forest plot of reoperation.

**Figure 5:**
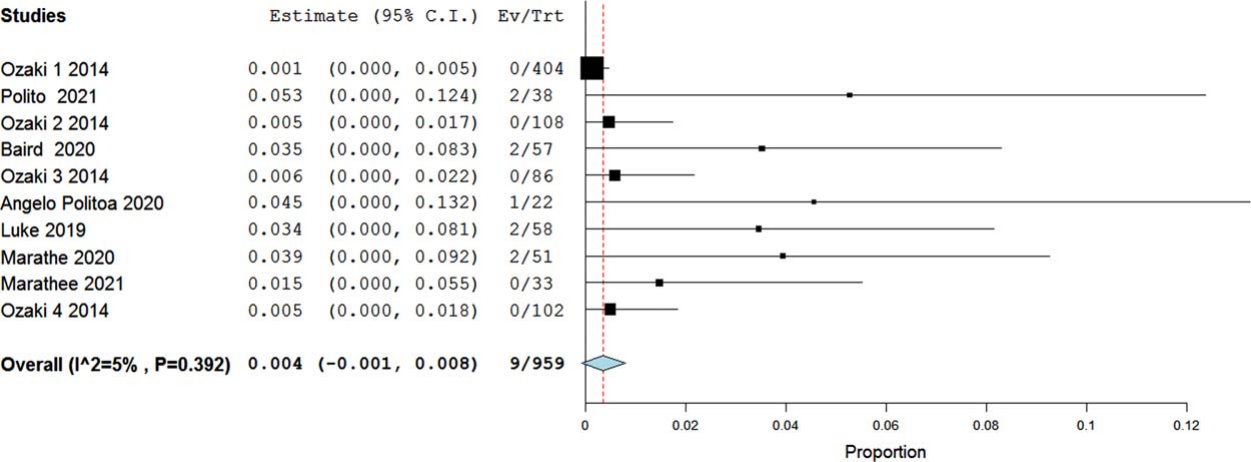
Forest plot of conversion to aortic valve replacement.

Our analysis of mean CPBT (min) included 18 studies, in which the Ozaki procedure showed a statistically significant pooled mean of 148 min (95% CI 144–152.2, *P*<0.001) (Fig. 2), and in the analysis of mean aortic cross-clamp time (min) (ACCT) 19 studies were included showing a mean time of 112.46 min (95% CI 105.116–119.823, *P*<0.001) (Fig. 3). Furthermore, the analysis of reoperation included 19 studies that showed a statistically significant low risk of reoperation with Ozaki operation as risk 0.011 (95% CI 0.005–0.016, *P*=0.047, *I*^2^=38%). Regarding conversion to AVR, an analysis including studies showed a low risk of conversion from the Ozaki procedure as risk 0.004 (95% CI −0.001–0.008, *P*=0.392, *I*^2^=5%) (Figs. 4, 5).

#### Postoperative outcomes analysis

**Figure 6:**
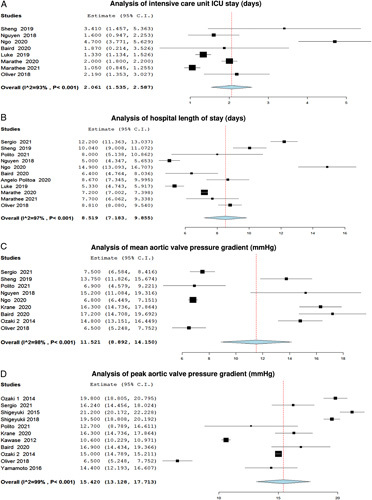
Forest plot of (A) ICU stay (days), (B) Hospital length of stay (days), (C) Mean aortic valve pressure gradient, and (D) Peak aortic valve pressure gradient (mmHg).

**Figure 7:**
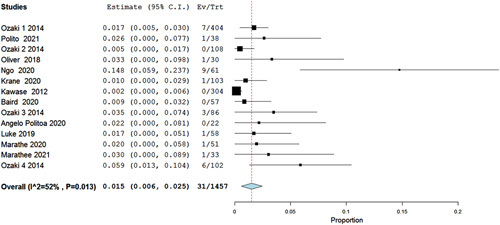
Forest plot of death.

We analyzed 8 and 11 studies for the ICU stay (days) and hospital length of stay (days) with the Ozaki operation, which showed a statistically significant pooled mean of 2.061 days (95% CI 1.535–2.587, *P*<0.001, *I*^2^=93%) and 8.159 days (95% CI 7.183–9.855, *P*<0.001, *I*^2^=97%), respectively. Moreover, the analysis of the mean and peak aortic valve pressure gradient (mmHg) showed a pooled mean of 11.521 (95% CI 8.892, 14.150, *P*<0.001, I2=98%) and 15.420 (95% CI 13.128–17.713, *P*<0.001, *I*^2^=99%), respectively (Fig. 6A–D). Regarding the risk of death, 14 studies were included showing a statistically significant low risk of death with the Ozaki procedure with a pooled risk of 0.015 (95% CI 0.006–0.025, *P*=0.013, *I*^2^=52%) (Fig. 7).

### Sensitivity analysis

Despite the significant heterogeneity that was observed in CPBT, cross-clamp time, ICU stay, hospital stay analyses, mean, and peak pressure gradient, (*I*^2^=92%), (*I*^2^=99%), (*I*^2^=93%), (*I*^2^=97%), (*I*^2^=98%), and (*I*^2^=99%), respectively, the leave-one-out analyses revealed that there were no single study effects on the overall effect size (Supplementary Figs. 1–8, Supplemental Digital Content 5, http://links.lww.com/MS9/A178, Supplemental Digital Content 6, http://links.lww.com/MS9/A179, Supplemental Digital Content 7, http://links.lww.com/MS9/A180, Supplemental Digital Content 8, http://links.lww.com/MS9/A181, Supplemental Digital Content 9, http://links.lww.com/MS9/A182, Supplemental Digital Content 10, http://links.lww.com/MS9/A183, Supplemental Digital Content 11, http://links.lww.com/MS9/A184, Supplemental Digital Content 12, http://links.lww.com/MS9/A185). However, Ngo 2020 in the ICU stay analysis was revealed to affect the overall effect size (Supplementary Figure 4, Supplemental Digital Content 8, http://links.lww.com/MS9/A181).

## Discussion

To the best of our knowledge, this meta-analysis is the largest to assess the efficacy of the Ozaki procedure in AVR. Our analysis for mean CPBT showed a statistically significant pooled mean of 148 min from included 18 studies, and a statistically significant pooled mean of 112.46 min for mean ACCT from 19 studies. Furthermore, the Ozaki procedure showed a statistically significant decreased risk of both reoperation with risk of 0.011, and conversion to AVR with risk of 0.004. The mean hospital length of stay (days) with Ozaki operation showed a statistically significant pooled mean of 2.061 days, and the mean and peak aortic valve pressure gradient (mmHg) showed a pooled mean of 11.521 and 15.420, respectively. We also observed a significantly decreased risk of death with the Ozaki procedure with a pooled risk of 0.015.

Recently, it has been a dilemma to choose between MV prosthesis and bioprosthetic valves (BV). This dilemma arises from the need for the long-term use of anticoagulants for the MVs group and therefore the increased risk of bleeding, especially in the elderly group. On the other hand, the bioprosthetic valve, although not needing anticoagulants, has an increased risk of reoperation due to its low durability. However, the incidence of bleeding after both types is another conflict. A recent study by Tosello *et al.* reported that about 20% of patients undergoing AVR develop atrial fibrillation during the period of follow-up, rendering them prone to using anticoagulants irrespective of the type of valve transplanted^12^.

In Leviner’s meta-analysis, which compared MV replacement versus bioprosthetic ones in 16 876 patients, they showed that BVs are associated with increased mortality risk by 22%, increased reoperation risk by 202%, and a 42% risk reduction of bleeding^13^. A similar SR done by Tasoudis *et al*. showed that mortality risk from either BVs or MVs differs according to age category. BVs show increased mortality risk in patients aged 50–70 years while they have a better survival rate in patients aged greater than 70 years and no difference between both types in patients less than 50 years^14^. Because of this dilemma, multiple studies were conducted and, consequently, the guidelines have been a dynamic change. From 2007 to 2017, ESC guidelines for valvular heart disease lowered the age limit recommended for MVs to less than 60 years for the aortic position based on type C evidence^15^. Clinical practice has shown another decrease in this age limit, showing that most patients aged between 50 and 69 years preferred the BVs, resulting in a wide age range using BVs^16–18^.

The increased age range for BVs usage in the current era may be attributed to different factors, including the illusion perceived by the patients that BVs are better than MVs as the bleeding outcome after MV transplantation is early witnessed postoperatively affecting the lifestyle of patients, while the main concern of BVs, which is reoperation is witnessed later^19^. The introduction of minimally invasive procedures such as valve-in-valve transcatheter aortic valve replacement (ViV TAVR)^20^. Recently, ViV TAVR is an alternative to resurgical aortic valve replacement for patients with bioprosthetic valve degeneration; it is a less invasive procedure that has more favorable outcomes than resurgical aortic valve replacement as the second operation is associated with increased surgical risk^21,22^. Bruno *et al.* showed that ViV TAVR has better short-term outcomes as regards major bleeding, new-onset AF, and length of hospital stay. However, there is no significant difference between both groups in the mid-term follow-up^23^.

According to the previously mentioned, studies discussing the pros and cons of bioprostheses are crucial. There are different types of bioprostheses including pericardial valves either autologous or xeno-grafts and pulmonary autografts (Ross procedure). The Ross procedure is considered the gold standard in children and young adults due to its good hemodynamic performance and growth potential^24–28^. However, there are some concerns regarding it such as fear of failure, especially if there was AI and potential dilatation of the graft that results from mechanical adaptation phenomena which are called ‘elastic fiber fragmentation’^29^. There are multiple generations of xeno-grafts including Trifecta, the Carpentier-Edwards Magna Ease, Mitroflow, Sorin’s Freedom Solo, and Freestyle. Those xeno-grafts are bovine pericardial bioprostheses that have the advantage of their fast application and they mimic the natural valve. On the other hand, the xeno-grafts have the risk of structural valve deterioration^30,31^, and they need aortic annulus enlargement due to their large size^32^. Many studies compared the subtypes of xeno-grafts with each other^33–36^.

Aortic Valve Neo-cuspidization (AVNeo) is the usage of the autologous pericardium in AVR. It has an advantage over the other BVs that it has better hemodynamics and a lower trans-valvular gradient. It was first reported by Duran *et al.* ^37–40^; that they used the autologous pericardium in augmentation of the native valve and thus increasing the coaptation zone after treatment of the pericardium with glutaraldehyde solution. This solution increases the resistance of the valves against degeneration and preserves their pliability. Another way of AVNeo using the autologous pericardium was prescribed by Halees *et al.* ^41^; they replaced the three aortic valves with a single pericardial strip. Ozaki *et al.* came up with a new technique in which they made up separate pericardial leaflets for each coronary cusp. Ozaki *et al.* ^42^ showed that their technique is especially effective in the treatment of calcified AS and in patients with small aortic annulus.

To the best of our knowledge, our systematic review and meta-analysis is the most comprehensive one to date discussing the Ozaki technique in AVR. There is only one SR and meta-analysis that was done by Benedetto *et al.* ^43^ as regards the AVNeo procedure. Benedetto *et al.* discussed the xeno-grafts and autologous pericardial grafts. However, we handled the Ozaki technique conclusively in a more comprehensive way; we included 22 studies with 2863 patients in contrast to 8 studies with a total number of patients equal to 1259 in Benedetto’s SR. Nevertheless, the AVNeo procedure in general and specifically the Ozaki technique is a substantial research question that lacks steady evidence and needs to be targeted by further comparative and randomized clinical trials in the future as most currently available evidence is just observational and single-arm studies.

Our study is the largest to further assess the effectiveness of such a newly proposed surgical technique, yet our main limitations come from the observational nature of all our included studies, besides the absence of a certain comparator in nearly all the included studies, thus, we conducted a single-arm meta-analysis which is not considered as powerful as double-arm. Although there was heterogeneity found in some of our outcomes, it was solved using sensitivity analysis and leave on out analysis to ensure the robustness of our analysis and explore the effect of each study separately, which showed no study affecting the pooled effect estimate. Moreover, our heterogeneity can be raised from the disparity in the baseline characteristics of operated patients as Ozaki is not secured for a specific population yet most of centers it is merely based on surgeons’ decision and whether other procedures are possible, with both the novelty and complexity of such a surgical technique making the surgical expertise as well a key role in the outcomes of each study.

To conclude, the Ozaki procedure provides a safe surgical technique with less mean CPBT and mean ACCT. Associated with a small mean postoperative hospital stay of 2 days, the Ozaki procedure has been associated with a low risk not only of reoperation but also of conversion to other AVR modalities or reoperation.

## Ethical approval

Our research is systemic review and meta-analysis. As a general rule, systematic reviews do not typically require ethical approval since they do not involve direct contact with human subjects.

## Informed consent statement

Not applicable.

## Sources of funding

No funding was received.

## Author contribution

A.K.A., M.S.A., E.R.G., and R.A.F. performed the screening and data extraction; A.K.A did the analysis; M.S.A., R.A.F., and E.R.G. wrote the primary draft, which was further edited, modified, and reviewed by A.K.A., R.A.F., and A.S.H. All authors reviewed and agreed to the final version of the manuscript. All the authors gave final approval of the version to be submitted.

## Conflicts of interest disclosure

The authors declare that there is no conflicts of interest.

## Research registration unique identifying number (UIN)

1. Name of the registry: not applicable.
2. Unique Identifying number or registration ID: not applicable.
3. Hyperlink to your specific registration (must be publicly accessible and will be checked): not applicable.

## Data availability statement

All data and figures are included in the manuscript and the supplementary data.

## Provenance and peer review

Not commissioned, externally peer-reviewed.

## Institutional review board (IRB) approval

Not applicable.
